# An Attempt to Characterize the Ciguatoxin Profile in *Seriola fasciata* Causing Ciguatera Fish Poisoning in Macaronesia

**DOI:** 10.3390/toxins11040221

**Published:** 2019-04-13

**Authors:** Pablo Estevez, David Castro, Ana Pequeño-Valtierra, José M. Leao, Oscar Vilariño, Jorge Diogène, Ana Gago-Martínez

**Affiliations:** 1 University of Vigo, Department of Analytical and Food Chemistry, Campus Universitario de Vigo, 36310 Vigo, Spain; paestevez@uvigo.es (P.E.); dcastro@uvigo.es (D.C.); apequeno@uvigo.es (A.P.-V.); leao@uvigo.es (J.M.L.); ovilarino@uvigo.es (O.V.); 2 European Union Reference Laboratory for Marine Biotoxins, CITEXVI, Campus Universitario de Vigo, 36310 Vigo, Spain; 3 IRTA, Marine and Continental Waters, Ctra. Poble Nou, km. 5.5, E-43540 Sant Carles de la Ràpita, Spain; jorge.diogene@irta.cat

**Keywords:** ciguatera fish poisoning, macaronesia, caribbean ciguatoxins, LC-MS/MS, N2a

## Abstract

Ciguatera Fish Poisoning is a worldwide concern caused by the consumption of fish contaminated with ciguatoxins not only in endemic regions in the Pacific Ocean or the Caribbean Sea but also in emerging areas of Macaronesia on the eastern Atlantic. The recent emergence of these toxins in other coastal areas worldwide, prompted the need for the characterization of the risk in these areas. This Ciguatera Fish Poisoning risk has been recently identified as a potential threat in subtropical areas of the Atlantic coast and scientific efforts are being focused in the identification and confirmation of the toxins involved in this potential risk. Neuroblastoma cell assay has been widely used for the evaluation of the toxicity in several marine biotoxin groups, and found to be a very useful tool for toxicity screening. LC-MS/MS has been also used for confirmatory purposes although the main limitation of the advances on LC-MS/MS development is due to commercial unavailability of reference materials and hampers method implementation and validation or even confirmation of the ciguatoxins (CTXs) responsible for the toxic profiles. While neuroblastoma cell assay (N2a) is typically used for toxicity screening as mentioned above, being necessary to confirm this N2a toxicity by LC-MS/MS, this study is designed using N2a as a tool to confirm the toxicity of the fractions obtained corresponding to potential CTXs analogues according to the analysis by LC-MS/MS. With this aim, an amberjack sample (*Seriola fasciata*) from Selvagen Islads (Portugal) and implicated in Ciguatera Fish Poisoning was analyzed by LC-MS/MS and Caribbean Ciguatoxins were found to be mainly responsible for the toxicity. N2a was used in this work as a tool to help in the confirmation of the toxicity of fractions obtained by HPLC. Caribbean Ciguatoxin-1 was found as the main analogue responsible for the N2a toxicity while three Caribbean Ciguatoxin-1 (C-CTX1) metabolites which contribute to the total toxicity were also identified.

## 1. Introduction

Ciguatera Fish Poisoning (CFP) is known as a food intoxication with incidents typically known in certain tropical and subtropical areas. CFP is endemic in regions of the Pacific Ocean and Caribbean Sea and associated with the consumption of fish contaminated with ciguatoxins (CTXs). CTXs are lipophilic, ladder-like cyclic polyethers and stable to temperature [1,2]. They are oxidation products resulting from fish metabolization of its algal precursors, which are produced by benthic dinoflagellates (*Gambierdiscus* spp. and *Fukuyoa* spp.) [3,4]. The lack of reference materials commercially available is the main limitation to advance, not only in methods implementation, but also in the toxicological characterization of the different CTX analogues involved in this contamination. On the other hand, the low levels of CTXs typically found in the fish tissue makes their analysis very challenging, even when applying very sensitive techniques [5]. Among the three different groups of ciguatoxins currently known (Pacific, Caribbean and Indian), Pacific ciguatoxins (P-CTXs) have been better characterized since they are most widely distributed and evaluated [6,7,8]. More than 21 different analogues have been already identified as responsible for the CFP in the Pacific areas, and significant progress has been recently made on the identification of different P-CTXs profiles depending on the geographical regions where these toxins occur [9,10,11].

Studies carried out in contaminated fish from the area of the Caribbean Sea have shown that Caribbean Ciguatoxin-1 and -2 (C-CTX1 and C-CTX2) are the main analogues responsible for the CTX profile [12]. These CTXs were first isolated from a Horse-eye jack (*Caranx latus*) and structurally characterized by Nuclear Magnetic Resonance [13,14]. Due to the limitations above mentioned regarding the lack of reference materials, studies focused on this group of toxins are still limited. A limited number of publications refer to fish contaminations associated to Caribbean CTX profiles. Fewer investigations have been carried out on C-CTXs, and most of them are just concentrated in fish from the Caribbean Sea [15,16]. Important efforts have been made to identify the toxins responsible for fish contaminations in the Caribbean Sea (French West Indies) and at least 12 different C-CTXs analogues have been already detected in fish from these areas [17,18] but no significant investigations have been carried out to characterize and confirm the presence of these C-CTXs analogues. The emergence of CTXs in European waters, in particular in eastern Atlantic areas, has been investigated and an opinion about these toxins has been also published by the European Food Safety Authority (EFSA) [19]. The risk evaluation has been mainly focused on Macaronesia, including the Canary Islands (Spain) and Madeira (Portugal). Some authors reported the presence of Pacific ciguatoxins (P-CTXs) [20] but the identification of Caribbean ciguatoxins in the Canary Islands has been also reported [21]. The characterization of the risk in these areas became a concern and investigations are currently in place to evaluate to which extent CFP represents a public health risk that needs to be controlled. The authors of this work are presently involved in a research project cofounded by the EFSA and coordinated by the Spanish Food Safety Agency (AESAN). The project is focused on the characterization of the risk of ciguatera fish poisoning in Europe, with particular emphasis in those EU areas from the eastern Atlantic where the problem had been already reported. The most recent results obtained on the characterization of the risk in the Canary Islands and Madeira, show that C-CTX1 seems to be the main analogue responsible of the contamination in this geographical region, although the presence of other C-CTX analogues is also suspected [22]. The main limitation to advance the characterization of CTXs is the lack of reference materials commercially available and in particular for C-CTXs. Despite this limitation, the support of collaborators working in this field, have made it possible to identify C-CTX1 as the main analogue responsible for the CFP contamination. However, the concentrations of this toxin in the samples analyzed so far are typically very low, which also limits the progress on the isolation of this toxin to be able to prepare pure standards, as well as the identification of possible analogues involved in CFP contamination. Based on these limitations and with the aim of achieving the main objectives of this project, a contingency plan has been established to prepare at least laboratory reference materials with known toxin profiles. The main objective of this work was to try to advance the characterization of the CTX profiles of samples from the areas above mentioned, and to achieve our goal we have used, not only our own materials, obtained through the EUROCIGUA project, which consisted of contaminated fish samples from Canary Islands and Madeira, but also trying to find materials that were used in the past from the areas described above. The analytical methods used for the screening and semi-quantitation of the toxicity were: a neuroblastoma cell assay (N2a) based on the action mode of CTXs on sodium channels; and for the further confirmation and full quantitation LC-MS/MS, which had been initially described by [23] and further optimized by [24] 

A very interesting and valuable sample that was kept under appropriate conditions, to avoid decomposition (−20 °C) at the European Union Reference Laboratory for Marine Biotoxins (EURLMB), which had been analyzed by [25], was selected to continue with the objective of profile characterization. The sample consisted of a fish species of amberjack (*Seriola fasciata*) implicated in a CFP. The fish was captured in Selvagen Islands (Madeira, Portugal) and consumed in a restaurant in Tenerife (Canary Islands, Spain). The sample exhibited high cytotoxicity by N2a assay [25]. The sample was recently reanalyzed by N2a for the screening of the CTX-like toxicity and further confirmation by LC-MS/MS. Once the high CTX toxicity was confirmed, the sample was selected for further cigutatoxin profile characterization. The approach used to achieve our objective was to first develop a HPLC fractionation, combined with the N2a cytotoxicity evaluation of the fractions, and further confirmation of the toxicity by characterizing the possible analogues implicated in this toxicity by LC-MS/MS under the optimized method described by [24].

## 2. Results

### 2.1. Cytotoxicity Assay

**Figure 1:**
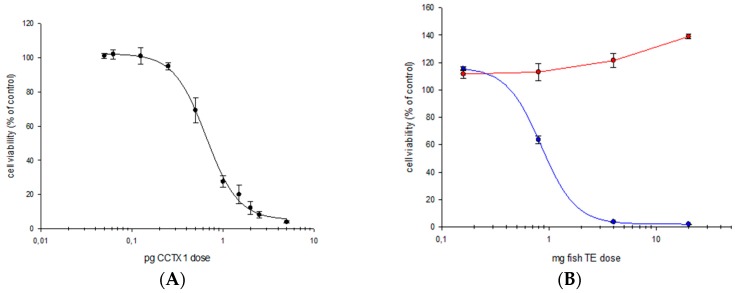
Neuroblastoma cell assay (N2a) cytotoxicity plots of (**A**) Caribbean Ciguatoxin-1 (C-CTX1) standard (**B**) amberjack (*Seriola fasciata*): blue line, sample treated with oubaine-veratridine; red line, sample without the oubaine-veratridine treatment.

The cytotoxicity of the purified extract of the contaminated fish was evaluated by N2a with the aim of evaluating the CTX-like response of this extract. A toxic response was observed in the treated oubaine-veratridine N2a cells whereas no positive response was detected in untreated cells confirming the presence of sodium channel specific activators (Figure 1). The semi-quantitation by N2a assay allows to determine a composite toxicity of 1.4 ng C-CTX1 eq·g^−1^ which is 14-fold above the guidance level proposed by the FDA, USA for C-CTX1 (0.1 ng·g^−1^) [26]. 

### 2.2. LC-MS/MS Analysis 

**Figure 2:**
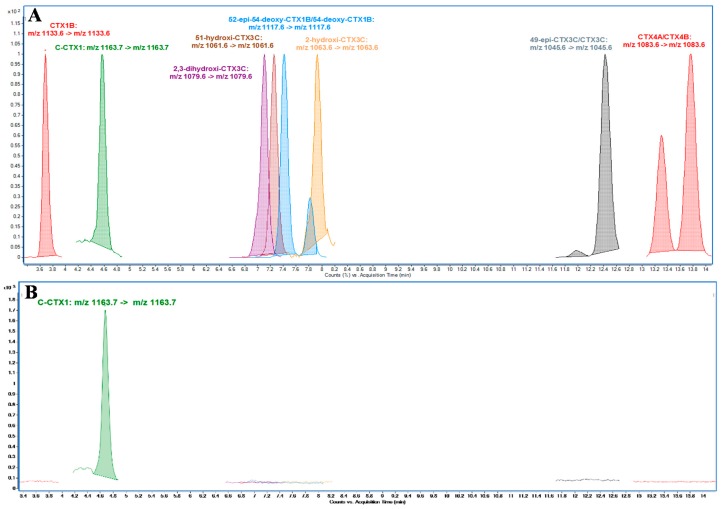
LC-MS/MS (MRM) analysis monitoring sodium adduct as precursor and product ion in (**A**) qualitative mixture of Pacific ciguatoxins (P-CTXs) and C-CTX1 standard (**B**) amberjack (*S. fasciata*) extract.

Previous studies carried out by this research group [24] showed an approach for the identification followed by confirmation of CTXs in the absence of reference materials, and is briefly described as follows. Identification is based on the high sensitivity detection of the sodium adducts and confirmation is by monitoring ions generated by several water losses and specific fragments. This approach has been used for C-CTXs, in particular C-CTX1, which have been recently identified as the main analogue responsible for the CFP in these areas [22,24]. The possible presence of Pacific ciguatoxins (P-CTXs) was also explored by using non-certified standards for 10 different P-CTXs analogues (kindly provided by Prof. Yasumoto). The results obtained showed that C-CTX1 was the only analogue clearly detected in this sample since the retention time and precursor/product ion transition, *m*/*z* 1163.7 -> *m*/*z* 1163.7, were consistent with those obtained for the C-CTX1 pure standard (kindly provided by Dr. R. Dickey and Dr. R. Manger) (Figure 2). Calibration curve was carried out with CTX1B standard due to the lack of enough C-CTX1 standard to obtain a proper quantitation (0.6–22.2 ng·mL^−1^, *R*^2^ = 0.999, *n* = 5). C-CTX1 standard equivalents were obtained by interpolating in CTX1B calibration. C-CTX1 in sample was quantified as CTX1B and converted to C-CTX1 with the equivalents previously obtained. The concentration of C-CTX1 in the fish sample evaluated in this study was 0.84 ng C-CTX1 ·g^−1^.

### 2.3. Sample Fractionation for CTX Profile Identification

**Figure 3:**
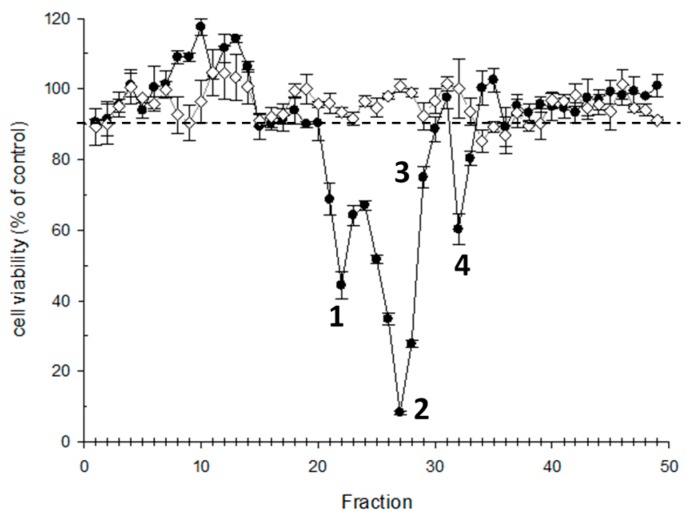
N2a cytotoxicity profile of HPLC fractionated amberjack sample: 4 mg of fish tissue equivalent (TE)/well.

The purified extract obtained after a double solid phase extraction (SPE) (Florisil + C18) according to [24] and equivalent to 100 g fish tissue was further fractionated by using HPLC. Fractions (49) collected were examined for cytotoxicity by N2a assay. The N2a cytotoxicity profile shows three prominent cytotoxic peaks (1,2 and 4) between the fraction 19 and 33 (Figure 3).

The fractions exhibiting cytotoxicity were analyzed by LC-MS/MS Multiple Reaction Monitoring (MRM) method monitoring several water losses [M+H-nH_2_O]^+^, sodium [M+Na]^+^ and a potassium [M+K]^+^ adducts and C-CTX1 specific fragments *m*/*z* 191.1, *m*/*z* 108.9. These two specific fragments were firstly described by [24] after the product ion analysis of C-CTX1 standard at high collision energy and were assigned to characteristic fragments of C-CTX1. The comparison of the ion ratios obtained with the C-CTX1 pure standard allows the confirmation of the possible C-CTXs analogues present in these toxic fractions (Table 1 and Table 2). 

**Figure 4:**
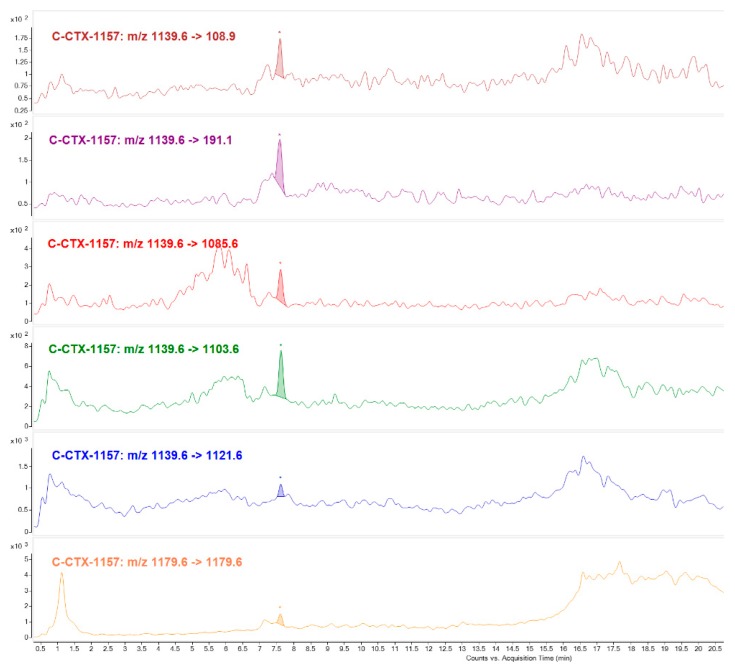
LC-MS/MS (MRM) analysis of Fraction 22 identifying putative C-CTX-1157 as responsible for the toxicity in N2a Toxic peak 1.

The positive CTX-like response obtained by N2a for the Fraction 22 (Toxic peak 1) might be attributed to the presence of an ion [M+H]^+^ *m*/*z* 1157.6, which highest intensity corresponds to the first water loss [M+H-H_2_O]^+^ *m*/*z* 1139.6. A cyclic polyether fragmentation was also observed detecting different water losses [M+H-nH_2_O]^+^ and also adducts formation [M+Na]^+^, [M+K]^+^. Further MRM analysis was carried out selecting the ion with the highest intensity [M+H-H_2_O]^+^ *m*/*z* 1139.6 and monitoring three additional water losses. [M+Na]^+^ was monitored as precursor and product ion at high collision energy, and C-CTX1 specific fragments (*m*/*z* 191.1 *m*/*z* 108.9) were used for final confirmation which allowed to conclude that the toxicity of the fraction analyzed might correspond to a C-CTX analogue different than C-CTX1 but structurally similar (Figure 4).

**Figure 5:**
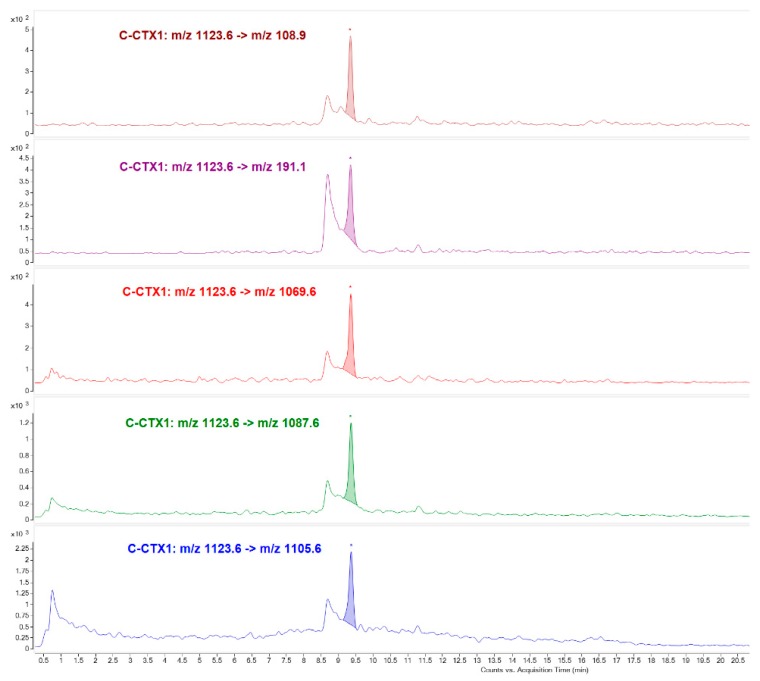
LC-MS/MS (MRM) analysis of Fraction 27 identifying C-CTX1 as responsible for the toxicity in N2a Toxic peak 2.

Fraction 27 (Toxic peak 2) was undoubtedly attributed to C-CTX1 since the availability of a pure standard for this particular compound allowed to establish its identity based, not only on the matching retention time, but also on the ion ratios for the different transitions used for confirmation (Figure 5).

**Figure 6:**
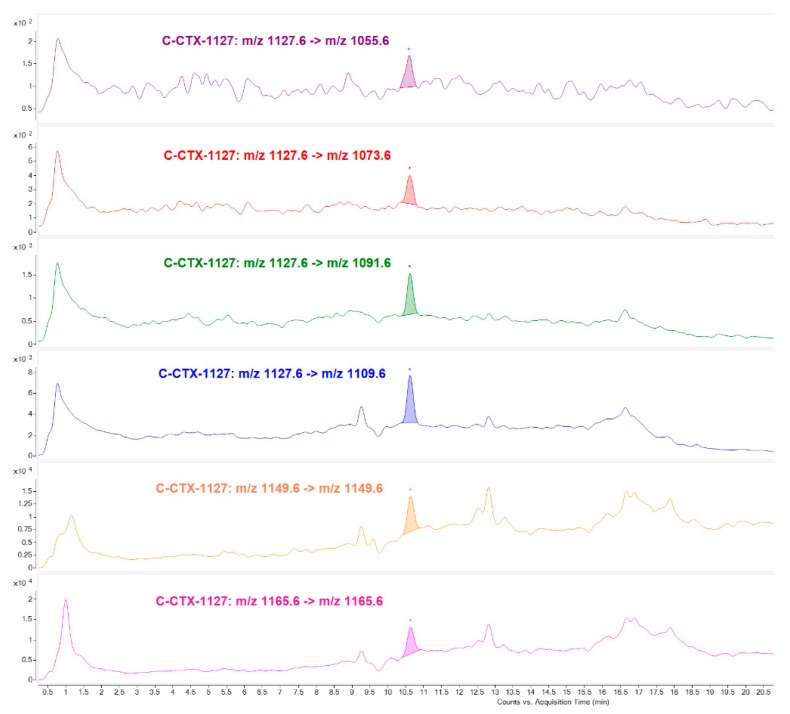
LC-MS/MS (MRM) analysis of Fraction 29 identifying putative C-CTX-1127 as responsible for the toxicity in N2a Toxic region 3.

In addition LC-MS/MS allows to identify in Fraction 29 a compound with [M+H]^+^ *m*/*z* 1127.6 which has not been clearly identified in the fraction analyzed by N2a (Region 3 of Figure 3). A cyclic polyether fragmentation was also observed and the further MRM analysis allowed to monitor up to four water losses as well as the [M+Na]^+^ adduct as precursor and product ion at high collision energy. Specific C-CTX1 fragments were not detected and the ion ratios were not consistent with that obtained in the standard, suggesting structural differences comparing with C-CTX1 (Figure 6).

**Figure 7:**
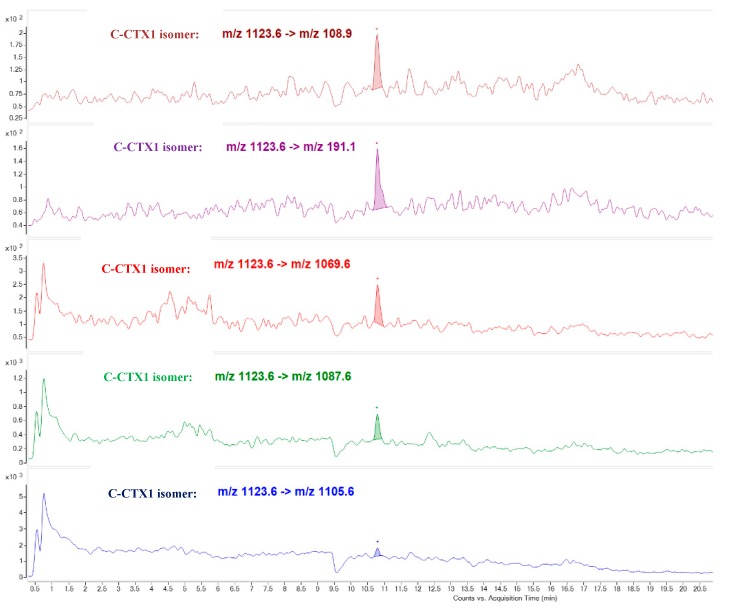
LC-MS/MS (MRM) analysis of Fraction 32 identifying putative C-CTX1 isomer as responsible for the toxicity in N2a Toxic peak 4.

The last eluting toxic compound detected in Fraction 32 (peak 4) was attributed to a C-CTX1 isomer due to the detection of the same ion transitions as that obtained in C-CTX1 pure standard but with a different retention time (Figure 7).

## 3. Discussion

To our knowledge this is the first report of the CTX profile of a fish from Macaronesia that is implicated in CFP. The absence of samples with very high concentration of CTXs from the Atlantic areas limits the progress on the characterization of the CTXs involved in CFP contamination. Therefore, it was considered that this fish species previously characterized as *S. fasciata* by genetic analysis which toxicity had been also confirmed by N2a (Sample #2 in the previous study) [25] showing a high CTX-like positive response, was considered to be a good candidate to be used for the characterization of the toxic profile. 

The present study was conducted using the CFP implicated tissue stored at −20 °C at the EURLMB. Sample was screened for cytotoxicity by N2a to ensure stability and the composite toxicity was determined as 1.4 ng C-CTX1 eq·g^−1^. N2a cytotoxicity assay and LC-MS/MS confirmatory analysis were performed on SPE purified extracts prepared following the same protocols. The concentration of C-CTX1 was determined by LC-MS/MS using the protocol previously reported [24] and the level found was 0.84 ng·g^−1^, which was lower than the composite sodium channel specific toxin level determined by N2a assay. LC-MS/MS analysis was focused on only C-CTX1 since it was reported as the major analogue responsible for the CTX toxicity in these areas [21,22,24].

The presence of P-CTXs was also evaluated using the P-CTXs standards available but none of these compounds were found in the sample analyzed, therefore the focus was on the Caribbean CTX profile, which supposed to be the one responsible for the toxicity of this sample, as previously mentioned. The approach of using the fractionation for evaluating the CTX-like toxicity by N2a on individual fractions is considered a very valuable tool to progress the characterization of the CTX profile, in the absence of standards for the CTX analogues. This approach also contributes to minimizing the possible misidentification due to the presence of interferences which could lead to a false conclusion about the CTX profile involved in the CFP contamination.

Full scan of the toxic fractions on the *m*/*z* range 1000–1200 did not provide any specific information about possible presence of cyclic polyether analogues since the sensitivity of this mode is not enough to reach the trace levels at which these analogues could be present, also taking into account that the matrix effect could have an impact on this detection. Due to these limitations, the approach used was focused on monitoring, by MRM, the CTX analogues suspected to be present, allowing a higher specificity and sensitivity as well as minimizing the matrix effects.

The results on the CTX analogues found in the evaluated sample, as well as the reasons that justify their characterization, are summarized as follows:

C-CTX1 and three C-CTX congeners of *m*/*z* 1157, *m*/*z* 1127 and *m*/*z* 1123 were detected in the HPLC purified fractions, with CTX-like activity by N2a. All these compounds produced characteristic ions associated with CTXs, including [M+H]^+^, [M+NH_4_]^+^, [M+Na]^+^, [M+K]^+^ and [M+H-nH_2_O]^+^.

C-CTX1 was identified mainly in Fraction 27, in the highest toxicity peak (Toxic peak 2) and considered to be mainly responsible for the total toxicity being appropriately identified due to the availability of C-CTX1 pure standard.

C-CTX congener of *m*/*z* 1157 was identified mostly in Fraction 22 (Toxic peak 1). Not only were water losses detected, selecting its higher intensity ion [M+H-H_2_O]^+^ *m*/*z* 1139 and sodium adduct [M+Na]^+^ *m*/*z* 1179 as precursor and product ion, but also C-CTX1 specific fragments (*m*/*z* 191.1 and *m*/*z* 108.9) with same ion ratios as C-CTX1 standard. This compound seems to be structurally similar to C-CTX1 in the ends of the molecule. C-CTX congener of *m*/*z* 1157 named as C-CTX-1157, was first identified by [17,18] in a horse-eye jack (*Caranx latus*) and barracuda (*Shyraena barracuda*) from French West Indies (Caribbean Sea) and more recently in a barracuda from the same geographical region [15]. Differing in 16 Da from C-CTX1, and with a retention time indicative of having a higher polarity, could suggest that this compound could be an oxidized product.

C-CTX congener of *m*/*z* 1127 was detected in Fraction 29 (Unresolved cytotoxic peak 3). Despite detecting the typical CTXs fragmentation pattern, ion ratios were not consistent with that obtained in C-CTX1 standard. This indicates structural differences compared to C-CTX1 which are also observed in the formation of its highest intensity ion [M+H]^+^ *m*/*z* 1127 in contrast with other C-CTXs congeners, e.g., putative C-CTX-1157 and C-CTX1 which its highest intensity ion is [M+H-H_2_O]^+^. Named as C-CTX-1127, this congener could be a demethylated product of C-CTX1 [15,18].

The last CTX congener identified seems to be a C-CTX1 isomer and it was detected mostly in Fraction 32 (Toxic peak 4). The same transitions and ion ratios as C-CTX1 were observed in LC-MS/MS (MRM) analysis, but with a higher retention in the HPLC-C18 column. The literature suggests that this compound might be C-CTX2, but the lack of pure standard did not allow identifying this CTX congener. This conclusion has been also reached by other authors [15,17].

Product ions of the different analogues at different collision energies did not show the formation of any prominent peaks. The low concentration of these congeners as well as the well-known high stability of CTXs chemical structure hampers the fragmentation and consequently the structural characterization of the obtained fragments. The limited availability of this material and the trace levels of CTXs did not allow further structural characterization or a quantitative study.

## 4. Conclusions

The approach used in this work combining the HPLC fractionation, the CTX-like total toxicity evaluation by N2a and the final characterization by LC-MS/MS allowed the identification of the CTXs profile responsible for the toxicity of the fish sample evaluated, also allowing to confirm previous results about the presence of Caribbean ciguatoxin as responsible for the CFP contamination in fish samples from the East Atlantic coast related to CFP. The results obtained in this work also allow to conclude that the CTX profile in this geographical region seems to be similar to the one reported for endemic areas of the Caribbean Sea.

## 5. Materials and Methods

### 5.1. Standards and Reagents

C-CTX1 pure standard solution (5 ng·mL^−1^) was kindly provided by Dr. Robert Dickey (previously, U.S. Food and Drug Administration) via Dr. Ronald Manger (Fred Hutchinson Cancer Research Center, Seattle, USA). A qualitative mixture of P-CTXs standard solution containing: CTX1B, 2,3-dihydroxyCTX3C, 51-hydroxyCTX3C, 52-*epi*-54-deoxyCTX1B/54-deoxyCTX1B, 2-hydroxyCTX3C, 49-*epi*CTX3C, CTX3C, CTX4A and CTX4B, was kindly provided by Prof. Takeshi Yasumoto (Japan Food Research Laboratories, Japan)

Acetone, diethyl ether, methanol, water, hexane and ethyl acetate used for extraction and purification were of HPLC grade (Merck KGaA, Darmstadt, Germany). Methanol, Acetonitrile, formic acid, ammonium formiate (Merck KGaA, Darmstadt, Germany) and water (J. T. Baker, Center Valley, PA, USA) for LC-MS analysis were of LC-MS grade. Methanol, formic acid, ammonium formiate (Merck KGaA, Darmstadt, Germany) for HPLC fractionation were of HPLC grade. Water for fractionation was deionized and purified at 15 MΩ·cm^−1^ through a Milli-Q Gradient A10 system (Millipore, Molsheim, France)

### 5.2. Samples

The sample used for this study consisted of a raw portion of a CFP-implicated amberjack (*Seriola fasciata*) supplied by EURLMB. The sample was provided to EURLMB by the Health Directory of Canary Islands (Tenerife). Amberjack fish of 37 kg was captured in Selvagen Islands (Portugal) and it was associated to CFP in 2008 in Tenerife (Spain) [25].

### 5.3. Sample Pretreatment

Sample pretreatment was carried out according to [24] conditions with some modifications. Briefly, 115 g of fish tissue was extracted twice with acetone (3 mL acetone/g fish tissue) (Ultra Turrax^®^ T25 basic IKA^®^ WERKE, Staufen, Germany) and centrifuged 10 min at 3000 rpm and 4 °C. The combined acetone layers were evaporated to an aqueous phase under reduced pressure (Syncore^®^ Polyvap, Barcelona, Spain). The remaining aqueous phase was extracted twice with diethyl ether (1 mL diethyl ether/g fish tissue) and the combined organic layers were dried under nitrogen steam. The organic residue was partitioned between 90% methanol (MeOH) and twice the volume of hexane (0.3 mL 90% MeOH/g fish tissue). The hexane layer was discarded evaporating the MeOH layer to a solid residue.

Solid residue from extraction was further purified by Solid Phase Extaction (SPE) (15 g fish tissue eq./cartridge). Conditions for SPE are briefly described as follows: Florisil SPE (J. T. Baker, 500 mg, Center Valley, PA, USA) was used to remove polar interfering compound, 2 mL of sample extract in ethyl acetate (AcOEt) was loaded in a cartridge previously conditioned with 3 mL of AcOEt, and eluted in three consecutive steps with 3 mL of AcOEt, 5 mL AcOEt:MeOH (9:1) and 5 mL of AcOEt:MeOH (3:1). CTXs eluted in the second elution were evaporated and further purified in C18 SPE (SUPELCLEAN, Supelco, 500 mg, Bellefonte, PA, USA). C18 cartridge was conditioned with 3 mL of 60% MeOH, Florisil SPE purified extract was dissolved in 2 mL of 60% MeOH and loaded in the cartridge, CTXs were retained and the cartridge was washed with 3 mL of 60% MeOH eluting with 5 mL of 90% MeOH.

A C18 purified extract of 15 g fish tissue eq. was evaporated and reconstituted in 0.5 mL of MeOH LC-MS. The extract was filtered (Syringe Driver filter Unit, Millex^®^-CV 0.22 um, 13 mm, Millipore, Billerica, MA, USA) prior to the analysis by both LC-MS/MS and N2a. The remaining C18 purified extracts equivalents to 100 g fish tissue were combined and evaporated to dryness to further fractionate in HPLC-C18.

### 5.4. HPLC-C18 Sample Fractionation

Sample fractionation was carried out on an Agilent 1100 G1312A binary pump coupled to an Agilent 1260 II fraction collector (Agilent Technologies, Waldbronn, Germany) with an Agilent 1260 II UV detector (Agilent Technologies, Waldbronn, Germany). Kinetex^®^ LC-C18 (4.6 × 250 mm, 5 µm, 100 A, Phenomenex) column was used for sample fractionation. The mobile phase for analysis consisted of: 5 mM ammonium formate and 0.1% formic acid in water (H_2_O) (A) and MeOH (B). Chromatographic separation was performed by a slow linear gradient elution: Starting with 60% B to 100% B over 85 min. The mobile phase flow rate was 1 mL·min^−1^ and the injection volume was 100 µL. Previous studies allowed to determine the chromatographic region where the CTX-like compounds elute optimizing the collection of the fractions in this region (data not shown). A total of 49 fractions were collected removing solvents under nitrogen. Cytotoxicity of each fraction was determined by N2a assay.

### 5.5. N2a Assay

Neuroblastoma 2-a cells (ATCC, CCL 131, Manassas, VA, USA) were cultured, maintained and used as described by [27,28] with slight modifications: incubation time was reduced to 16 h and the concentration of Oubain/Veratridine (O/V) (O3125/ V5754, Sigma, St. Louis, MO, USA) was adjusted to improve sensitivity. Semiquantitation to obtain composite toxicity was expressed as ng C-CTX1 equivalents ·g^−1^ fish tissue. Samples and C-CTX1 standard were analyzed in triplicate and 96-well plate (Corning Incorporated, Corning, NY, USA) format was used for the assay.

### 5.6. LC-MS/MS Analysis

An Agilent 1290 Infinity Liquid Chromatography system coupled to an Agilent 6495 Triple Quadrupole LC-MS (Agilent Technologies, Waldbronn, Germany) with iFunnel ionization was used to perform the LC-MS/MS analysis following the conditions described by [24]. Two different approaches were used:

A first method was used for CTXs identification and quantitation purposes: Poroshell 120 EC-C18 (3.0 × 50 mm, 2.7µm, Agilent, Waldbronn, Germany) column was used for CTXs separation at 40 °C. The mobile phase for analysis consisted of: 5 mM ammonium formate and 0.1% formic acid (Merck KGaA, Darmstadt, Germany) in water (H_2_O) (J. T. Baker, Center Valley, PA, USA) (A) and Methanol (MeOH) (Merck KGaA, Darmstadt, Germany) (B). Chromatographic separation was performed by gradient elution: Starting with 78% B to 88% B in 10 min holding for 5 min, increased to 100% B at 15.01 min for columns washing holding for 3 min reducing to initial conditions at 18 min equilibrating for 4 min before the next analysis. The mobile phase flow rate was 0.4 mL·min^−1^ and the injection volume was 1 µL. The MS method operated in positive ionization mode monitoring [M+Na]^+^ as precursor and product ions with collision energy of 40 eV. Drying gas was set at 15 L·min^−1^ of N_2_ at 290 °C; sheath gas flow, 12 L·min^−1^ of N_2_ at 400 °C; nebulizer gas, N_2_ at 50 psi; capillary voltage, 5000 V; nozzle voltage: 300 V; fragmentor potential 380 V. All analyses were performed in Multiple Reaction Monitoring (MRM) mode. CTXs were monitored as follows: CTX1B (*m*/*z* 1133.6), C-CTX1 (*m*/*z* 1163.7), 2,3-dihydroxyCTX3C (*m*/*z* 1079.6), 51-hydroxyCTX3C (*m*/*z* 1061.6), 52-*epi*-54-deoxyCTX1B/54-deoxyCTX1B (*m*/*z* 1117.6), 2-hydroxyCTX3C (*m*/*z* 1063.6), 49-*epi*CTX3C/CTX3C (*m*/*z* 1045.6), CTX4A/CTX4B (*m*/*z* 1083.6)

A second method was used for C-CTX1 confirmatory purposes and CTXs analogues identification: Poroshell 120 EC-C18 (2.1 × 100 mm, 2.7 μm, Agilent USA) column was used for CTXs separation at 40 °C. The mobile phase for analysis consisted of: 5 mM ammonium formate and 0.1% formic acid in H_2_O (A) and Acetonitrile (MeCN) (Merck KGaA, Darmstadt, Germany) (B). Chromatographic separation was performed by gradient elution: Starting with 35% B for 2 min, linear gradient to 80% B at 15 min, increased to a 95% B at 16 min, holding for 5 min and reducing afterward to 35% B at 24 min equilibrating for 4 min before the next analysis. The mobile phase flow rate was 0.4 mL·min^−1^ and the injection volume was 5 µL. The MS method operated in positive ionization mode monitoring water loss ions ([M+H-nH_2_O]^+^) and C-CTX1 fragments by MRM mode. MS/MS transitions, collision energy (CE) as well as Collision Acceleration Voltage (CAV) for each precursor/product ion are summarized in Table 2. Drying gas was set at 16 L·min^−1^ of N_2_ at 250 °C; sheath gas flow, 12 L·min^−1^ of N_2_ at 400 °C; nebulizer gas, N_2_ at 15 psi; capillary voltage, 5000 V; nozzle voltage: 1000 V; fragmentor potential 380 V.

## Figures and Tables

**Table 1:** Caribbean Ciguatoxins identified in N2a toxic fractions (n.d. not detected) *According to [17].

Peak	1	2	3	4
Fractions	19–22	23–28	29	32–33
Retention Time (min)	7.6	9.4	10.6	10.8
[M+K]^+^	1195.6	1179.6	1165.6	n.d.
[M+Na]^+^	1179.6	1163.6	1149.6	1163.6
[M+NH_4_]^+^	1174.6	1158.6	1144.6	n.d.
[M+H]^+^	1157.6	1141.6	1127.6	n.d.
[M+H-nH_2_O]^+^ n range	1–3	1–3	1–4	1–3
C-CTX identification *	C-CTX-1157	C-CTX1	C-CTX-1127	Unknown

**Table 2:** MS/MS conditions for the confirmation of the different C-CTXs analogues identified in an amberjack (*S. fasciata*) sample.

Compound	Precursor Ion (Q1)	Product Ion (Q3)	CE (eV)
**C-CTX-1157**	[M+Na]^+^ *m*/*z* 1179.6	[M+Na]^+^ *m*/*z* 1179.6	40
	[M+H-H_2_O]^+^ *m*/*z* 1139.6	[M+H-2H_2_O]^+^ *m*/*z* 1121.6	15
	[M+H-H_2_O]^+^ *m*/*z* 1139.6	[M+H-3H_2_O]^+^ *m*/*z* 1103.6	30
	[M+H-H_2_O]^+^ *m*/*z* 1139.6	[M+H-4H_2_O]^+^ *m*/*z* 1085.6	30
	[M+H-H_2_O]^+^ *m*/*z* 1139.6	*m*/*z* 191.1	41
	[M+H-H_2_O]^+^ *m*/*z* 1139.6	*m*/*z* 108.9	52
**C-CTX1 and isomers**	[M+H-H_2_O]^+^ *m*/*z* 1123.6	[M+H-2H_2_O]^+^ *m*/*z* 1105.6	25
	[M+H-H_2_O]^+^ *m*/*z* 1123.6	[M+H-3H_2_O]^+^ *m*/*z* 1087.6	29
	[M+H-H_2_O]^+^ *m*/*z* 1123.6	[M+H-4H_2_O]^+^ *m*/*z* 1069.6	37
	[M+H-H_2_O]^+^ *m*/*z* 1123.6	*m*/*z* 191.1	41
	[M+H-H_2_O]^+^ *m*/*z* 1123.6	*m*/*z* 108.9	52
**C-CTX-1127**	[M+Na]^+^ *m*/*z* 1149.6	[M+Na]^+^ *m*/*z* 1149.6	40
	[M+H]^+^ *m*/*z* 1127.6	[M+H-H_2_O]^+^ *m*/*z* 1109.6	30
	[M+H]^+^ *m*/*z* 1127.6	[M+H-2H_2_O]^+^ *m*/*z* 1091.6	32
	[M+H]^+^ *m*/*z* 1127.6	[M+H-3H_2_O]^+^ *m*/*z* 1073.6	37
	[M+H]^+^ *m*/*z* 1127.6	[M+H-4H_2_O]^+^ *m*/*z* 1055.6	40
